# A Comparative Study of Surgically Induced Astigmatism From Temporal and Steep-Axis Clear Corneal Incisions in Phacoemulsification Cataract Surgery

**DOI:** 10.7759/cureus.91714

**Published:** 2025-09-06

**Authors:** Rukshar S Mujawar, Umeshaben I Padvi, Arth Vasavada, Manali Shah, Akshay Chaudhari

**Affiliations:** 1 Ophthalmology, Gujarat Medical Education & Research Society (GMERS) Medical College and Hospital, Valsad, IND

**Keywords:** corneal astigmatism, phacoemulsification, steep-axis incision, surgically induced astigmatism, temporal incision

## Abstract

Background

Surgically induced astigmatism (SIA) is a critical determinant of visual outcomes after phacoemulsification cataract surgery. The choice of incision site significantly influences postoperative refractive stability. This study compares temporal clear corneal incision (CCI) and steep-axis CCI in terms of SIA, uncorrected visual acuity (UCVA), and corneal stability.

Methods

This prospective randomized study included 130 patients with senile cataract and preoperative corneal astigmatism ≤1.5 D (diopters), allocated into two groups: Group A (temporal incision, n = 65) and Group B (steep-axis incision, n = 65). All surgeries were performed by a single surgeon using standardized phacoemulsification techniques. Preoperative and postoperative assessments included UCVA, best-corrected visual acuity (BCVA), corneal astigmatism, and SIA calculated using vector analysis (magnitude only). Follow-up was performed at day 1, day 7, and one month. Data were analyzed using independent t-tests and chi-square tests, with p < 0.05 considered significant.

Results

Baseline characteristics were comparable, though a minor imbalance in preoperative astigmatism was noted. At one month, UCVA was significantly better in the temporal group (p = 0.020), while BCVA showed no intergroup difference. Median SIA was lower with temporal incisions (p = 0.003). Corneal astigmatism was also reduced in Group A at day 7 (p < 0.001) and one month (p < 0.001). At one month, 93.8% of patients in Group A achieved corneal astigmatism ≤0.5 D versus 61.5% in Group B (p < 0.001). No significant differences were observed in with-the-rule (WTR) or against-the-rule (ATR) astigmatism patterns.

Conclusion

Temporal CCI induced less astigmatism and provided better early visual outcomes compared with steep-axis CCI. While these results support the temporal approach for optimizing refractive outcomes in cataract surgery, interpretation should be cautious given the short follow-up, lack of masking, and limited reporting of vector direction. Larger, long-term studies are warranted to validate these findings.

## Introduction

Cataract is the leading cause of reversible blindness globally, with an estimated 15 million individuals in India alone affected by untreated cataracts [1]. It is characterized by progressive clouding of the lens, primarily in older adults, with age as the most significant risk factor. The pathogenesis involves oxidative stress, ultraviolet radiation, and genetic predisposition [2]. Phacoemulsification with intraocular lens (IOL) implantation is the gold standard for cataract surgery due to its minimal invasiveness, faster visual recovery, and superior postoperative outcomes [3]. Despite these advantages, surgically induced astigmatism (SIA) remains a critical determinant of postoperative vision, especially for patients with pre-existing corneal astigmatism. Even low residual astigmatism (≥0.50 diopters (D)) can adversely affect uncorrected visual acuity (UCVA) and patient satisfaction [4].

Astigmatism in cataract surgery consists of two main components: preoperative astigmatism, intrinsic to the patient, and SIA, introduced by the surgical technique [5]. Corneal astigmatism may be with-the-rule (WTR) or against-the-rule (ATR), and modern surgical planning aims to minimize postoperative refractive error through strategic incision placement [6,7]. With the evolution of surgical techniques, cataract surgery has increasingly become a refractive procedure, where reducing SIA is equally important as removing the cataract itself.

Clear corneal incisions (CCIs) are widely used in phacoemulsification due to reduced postoperative inflammation, minimal SIA, and faster recovery compared to sclerocorneal and limbal incisions [8]. Their impact depends on design (single-plane, biplanar, or triplanar), as well as wound direction, depth, and width [9]. The incision site is particularly critical: temporal CCI, typically at 180°, is favored for its neutral effect on corneal curvature, lower average SIA (0.25-0.50 D), and ergonomic advantage for right-handed surgeons [10]. Steep-axis CCI, aligned with the patient’s preoperative steep meridian, has gained attention for potentially reducing pre-existing astigmatism. While some studies report greater flattening and effective correction, variability in SIA outcomes remains high [11]. Currently, no consensus exists on the superior approach, as outcomes depend on incision size, corneal biomechanics, and baseline astigmatism [12].

This study was undertaken to compare the effects of temporal versus steep-axis CCIs on SIA in phacoemulsification cataract surgery. The primary outcome was SIA. Secondary outcomes included UCVA, best-corrected visual acuity (BCVA), and postoperative corneal stability.

We hypothesized that temporal CCIs would induce lower and more predictable SIA due to their biomechanical neutrality, whereas steep-axis CCIs would demonstrate greater variability but potential benefit in reducing pre-existing corneal astigmatism.

## Materials and methods

Study design and setting

This hospital-based, prospective, observational, comparative study was conducted in the Department of Ophthalmology, Gujarat Medical Education & Research Society (GMERS) Medical College and Hospital, Valsad, India, from July 2022 to June 2024. Institutional Ethics Committee approval was obtained (MCV/IHEC/611; dated 15/07/2022), and written informed consent was obtained from all participants prior to enrollment.

Study population and sample size

Patients aged 40 to 90 years with clinically diagnosed immature senile cataract were included. Only those with pre-existing regular corneal astigmatism up to 1.5 D, either WTR or ATR, were eligible. Participants with no prior history of ocular surgery and those willing to provide written informed consent and comply with follow-up visits were included. Patients with irregular astigmatism, pre-existing astigmatism greater than 1.5 D, hard cataracts, corneal pathologies such as dystrophies, degenerations, pseudoexfoliation, or other ocular diseases - including glaucoma, macular or retinal diseases, diabetic retinopathy, pterygium, traumatic or complicated cataract, lenticular subluxation - or any systemic illness affecting visual outcomes were excluded.

The sample size was calculated a priori based on expected differences in SIA between temporal and steep-axis CCIs. Prior review by Pattanayak et al. has reported mean SIA differences of approximately 0.25-0.30 D, with a standard deviation of 0.5 D [6]. Assuming an effect size of 0.3 D, with 80% power and a two-sided alpha of 0.05, a minimum of 118 eyes was required. Allowing for 10% attrition, the final sample size was set at 130 eyes (65 eyes per group).

Randomization and allocation concealment

Eligible patients were randomly allocated into two groups: Group A (temporal CCI) and Group B (steep-axis CCI). Randomization was performed using a computer-generated random number table. Allocation concealment was maintained using sequentially numbered, opaque, sealed envelopes (SNOSE), which were prepared and managed by an independent staff member not otherwise involved in patient recruitment or surgery. The surgeon could not be blinded to the incision site; however, outcome assessments (keratometry and visual acuity measurements) were performed by masked examiners to minimize detection bias.

Preoperative assessment

After obtaining informed consent, all patients underwent a detailed preoperative assessment. Visual acuity was measured using a Snellen chart for literate patients and a Landolt’s C chart for illiterate patients [13], with results documented on the LogMAR scale. A comprehensive ophthalmologic evaluation was performed, including slit-lamp biomicroscopy for anterior segment examination; keratometry using a Bausch & Lomb keratometer (Bausch & Lomb Incorporated, Rochester, NY, USA) to classify astigmatism as WTR (60°-120°) or ATR (0°-20° or 150°-180°); and intraocular pressure measurement using a non-contact tonometer. IOL power was determined using A-scan ultrasound biometry with the SRK/T formula, and cataract severity was graded based on the Lens Opacities Classification System III (LOCS III) [14]. Routine preoperative investigations and a Xylocaine sensitivity test were conducted to ensure patient safety.

Surgical procedure

All surgeries were performed under peribulbar anesthesia by the same experienced surgeon to eliminate inter-surgeon variability. A standard phacoemulsification technique with foldable IOL implantation was employed. The ocular surface was disinfected with 5% povidone-iodine, and a sterile drape was applied. Digital ocular massage was performed for three to five minutes before surgery.

For Group A (temporal incision approach), the surgeon was positioned at 9 o’clock for the right eye and 3 o’clock for the left eye. A single-step, biplanar, self-sealing incision was made using a 2.8 mm keratome, approximately 0.5 mm inside the limbus. Two 1.0 mm side-port incisions were created at the 2 o’clock position relative to the main incision. The procedure included continuous curvilinear capsulorrhexis, hydrodissection, nucleus emulsification, cortical aspiration, and implantation of a foldable hydrophilic acrylic IOL. The corneal wounds were left sutureless, sealed with stromal hydration.

For Group B (steep-axis incision approach), the surgical steps were similar, but the primary incision was made along the steepest corneal meridian, with the surgeon positioned facing the steep axis. Side-port incisions were created in the same manner as in Group A.

Instrument calibration

Keratometry was performed using a Bausch & Lomb manual keratometer (Bausch & Lomb Incorporated), which was calibrated daily according to the manufacturer’s recommendations. Axial length measurements were obtained with an ultrasound A-scan biometer, calibrated before each session using a standard eye model.

Postoperative assessment and follow-up

The primary outcome was SIA, calculated by vector analysis. The Holladay-Cravy-Koch method of vector analysis was used for determining SIA, as it is widely accepted for precise quantification of astigmatic changes [7]. Secondary outcomes included UCVA, BCVA, and any intraoperative or postoperative complications. Assessments included UCVA and BCVA using Snellen’s chart. Postoperative evaluations were conducted at one week, one month, and six weeks.

Data collection and statistical analysis

Data collection was conducted using a pre-designed proforma. Data were analyzed using IBM SPSS Statistics for Windows, Version 20 (Released 2011; IBM Corp., Armonk, NY, USA). Continuous variables were summarized as means ± standard deviation (SD) for normally distributed data, or as median (interquartile range, or IQR) for non-normally distributed data. Categorical variables were expressed as frequencies and percentages. Normality was assessed using the Shapiro-Wilk test. Between-group comparisons were performed using independent t-tests (or Mann-Whitney U test for non-normal data), while within-group changes were analyzed with paired t-tests (or Wilcoxon signed-rank test, as appropriate). Chi-square or Fisher’s exact test was used for categorical variables. A p-value <0.05 was considered statistically significant. Holm correction was applied for multiple comparisons in the demographic ANOVA table.

## Results

A total of 130 patients were randomized, with 65 undergoing temporal incisions and 65 undergoing steep-axis incisions. The mean age of participants in Group A (temporal incision approach) and Group B (steep-axis incision approach) was similar (59.2 ± 9.02 vs. 59.4 ± 9.49 years, p = 0.887), and the age distribution across different age categories did not show a significant difference (p = 0.406). While Group B (steep-axis incision approach) had a higher proportion of female participants (39 out of 65, 60.0%) compared to Group A (temporal incision approach) (30 out of 65, 46.2%), the gender distribution was not statistically significant (p = 0.114) (Table 1).

**Table 1:** Baseline Characteristics of the Study Population

Variables	Group A (n = 65)	Group B (n = 65)	Test of significance	p-value
	Frequency (%)/Mean ± SD			
Age (in years)				
40-50	14 (21.5)	15 (23.1)	χ² = 2.914	0.406
51-60	20 (30.8)	22 (33.8)		
61-70	25 (38.5)	18 (27.7)		
>70	6 (9.2)	10 (15.4)		
Age (years)	59.2 ± 9.02	59.4 ± 9.49	t = 0.147	0.887
Gender				
Female	30 (46.2)	39 (60.0)	χ² = 2.494	0.114
Male	35 (53.8)	26 (40.0)		

Preoperatively, UCVA distribution was comparable between the groups (p = 0.477). On postoperative day 1, most patients in both groups achieved UCVA of 0.0-0.18 LogMAR: 48 out of 65 patients (73.8%) in Group A (temporal incision approach) and 50 out of 65 patients (76.9%) in Group B (steep-axis incision approach) (p = 0.46). By postoperative day 7, a significantly higher proportion of patients in Group B (60 out of 65, 92.3%) had UCVA of 0.0-0.18 LogMAR compared to Group A (59 out of 65, 90.8%) (p = 0.006). At one month, all patients in both groups (65 out of 65, 100.0%) achieved UCVA of 0.0-0.18 LogMAR, with no significant difference between them (p = 0.134) (Table 2).

**Table 2:** Distribution of UCVA (LogMAR) Over Time in Group A and Group B UCVA, Uncorrected Visual Acuity; LogMAR, Logarithm of the Minimum Angle of Resolution

UCVA (LogMAR)	Group A (n = 65)	Group B (n = 65)	Test of significance	p-value
	Frequency (%)			
Pre-OP				
0.0 & 0.18	0 (0.0)	0 (0.0)	χ² = 2.518	0.477
0.3 & 0.48	15 (23.1)	18 (27.7)		
0.6 & 0.78	49 (75.4)	44 (67.7)		
≥1.0	1 (1.5)	3 (4.6)		
Day 1				
0.0 & 0.18	48 (73.8)	50 (76.9)	χ² = 0.789	0.46
0.3 & 0.48	16 (24.6)	14 (21.5)		
0.6 & 0.78	1 (1.5)	1 (1.5)		
≥1.0	0 (0.0)	0 (0.0)		
Day 7				
0.0 & 0.18	59 (90.8)	60 (92.3)	χ² = 10.183	0.006
0.3 & 0.48	6 (9.2)	5 (7.7)		
0.6 & 0.78	0 (0.0)	0 (0.0)		
≥1.0	0 (0.0)	0 (0.0)		
1 Month				
0.0 & 0.18	65 (100.0)	65 (100.0)	Fisher-Freeman-Halton Exact = 3.34	0.134
0.3 & 0.48	0 (0.0)	0 (0.0)		
0.6 & 0.78	0 (0.0)	0 (0.0)		
≥1.0	0 (0.0)	0 (0.0)		

Preoperatively, Group B (steep-axis incision approach) had a higher proportion of patients with BCVA in the 0.3-0.48 LogMAR range (44 out of 65, 67.7%) compared to Group A (temporal incision approach) (33 out of 65, 50.8%), while Group A had more patients in the 0.6-0.78 LogMAR range (32 out of 65, 49.2%) compared to Group B (21 out of 65, 32.3%) (p = 0.05). On postoperative day 1, BCVA distribution was comparable between the groups (p = 0.917), with 0.0-0.18 LogMAR seen in 15 out of 65 (23.1%) in Group A and 10 out of 65 (15.4%) in Group B. By day 7, BCVA of 0.0-0.18 LogMAR was observed in 46 out of 65 (70.8%) in Group A and 30 out of 65 (46.2%) in Group B (p = 0.753). At one month, 0.0-0.18 LogMAR BCVA was recorded in 55 out of 65 patients (84.6%) in Group A and 47 out of 65 patients (72.3%) in Group B, though the difference was not statistically significant (p = 0.134) (Table 3).

**Table 3:** Distribution of BCVA (LogMAR) Over Time in Group A and Group B BCVA, Best-Corrected Visual Acuity; LogMAR, Logarithm of the Minimum Angle of Resolution

BCVA (LogMAR)	Group A (n = 65)	Group B (n = 65)	Test of significance	p-value
	Frequency (%)			
Pre-OP				
0.0 & 0.18	0 (0.0)	0 (0.0)	χ² = 5.985	0.05
0.3 & 0.48	33 (50.8)	44 (67.7)		
0.6 & 0.78	32 (49.2)	21 (32.3)		
≥1.0	0 (0.0)	0 (0.0)		
Day 1				
0.0 & 0.18	15 (23.1)	10 (15.4)	χ² = 0.519	0.917
0.3 & 0.48	40 (61.5)	43 (66.2)		
0.6 & 0.78	9 (13.8)	12 (18.5)		
≥1.0	1 (1.5)	0 (0.0)		
Day 7				
0.0 & 0.18	46 (70.8)	30 (46.2)	χ² = 0.572	0.753
0.3 & 0.48	19 (29.2)	32 (49.2)		
0.6 & 0.78	0 (0.0)	3 (4.6)		
≥1.0	0 (0.0)	0 (0.0)		
1 Month				
0.0 & 0.18	55 (84.6)	47 (72.3)	Fisher-Freeman-Halton Exact = 4.09	0.134
0.3 & 0.48	10 (15.4)	17 (26.2)		
0.6 & 0.78	0 (0.0)	1 (1.5)		
≥1.0	0 (0.0)	0 (0.0)		

UCVA, measured in LogMAR units, was significantly better in Group A (temporal incision approach) at postoperative day 7 (mean ± SD: 0.168 ± 0.146) compared to Group B (steep-axis incision approach) (0.253 ± 0.191, p = 0.01), and also at one month (0.077 ± 0.131 vs. 0.138 ± 0.167, p = 0.02). BCVA, also measured in LogMAR, did not show a statistically significant difference between the groups at any postoperative time point (p > 0.05). SIA, measured in diopters (D), was significantly lower in Group A at one month (0.94 ± 0.445 D) compared to Group B (1.23 ± 0.628 D, p = 0.003). Corneal astigmatism was also significantly lower in Group A at both day 7 (0.49 ± 0.278 D vs. 0.71 ± 0.378 D, p < 0.001) and one month (0.35 ± 0.215 D vs. 0.61 ± 0.417 D, p < 0.001), indicating a more favorable astigmatic outcome with the temporal incision approach (Table 4).

**Table 4:** Comparison of Visual Acuity, Surgically Induced Astigmatism, and Corneal Astigmatism Between Group A and Group B Over Time UCVA, Uncorrected Visual Acuity; BCVA, Best-Corrected Visual Acuity; SIA, Surgically Induced Astigmatism; LogMAR, Logarithm of the Minimum Angle of Resolution

Variables	Group A (n = 65)	Group B (n = 65)	Test of significance	p-value
	Mean ± SD			
UCVA (LogMAR)				
Pre-OP	0.776 ± 0.256	0.742 ± 0.266	t = 0.741	0.460
Post-OP Day 1	0.378 ± 0.185	0.412 ± 0.159	t = 1.134	0.260
Post-OP Day 7	0.168 ± 0.146	0.253 ± 0.191	t = 2.587	0.010
Post-OP Month 1	0.077 ± 0.131	0.138 ± 0.167	t = 2.348	0.020
BCVA (LogMAR)				
Pre-OP	0.528 ± 0.223	0.441 ± 0.254	t = 2.062	0.040
Post-OP Day 1	0.180 ± 0.146	0.143 ± 0.137	t = 0.914	0.367
Post-OP Day 7	0.077 ± 0.107	0.059 ± 0.100	t = 0.367	0.721
Post-OP Month 1	0.008 ± 0.038	0.000 ± 0.000	t = 1.661	0.100
SIA (D)				
Pre-OP	0.51 ± 0.312	0.53 ± 0.298	t = 0.427	0.674
Post-OP Day 1	0.61 ± 0.412	0.69 ± 0.529	t = 0.965	0.340
Post-OP Day 7	1.04 ± 0.490	1.73 ± 3.584	t = 1.564	0.122
Post-OP 1 Month	0.94 ± 0.445	1.23 ± 0.628	t = 3.092	0.003
Corneal Astigmatism (D)				
Pre-OP	0.62 ± 0.386	0.71 ± 0.433	t = 1.238	0.221
Post-OP Day 1	0.68 ± 0.400	0.78 ± 0.419	t = 1.424	0.160
Post-OP Day 7	0.49 ± 0.278	0.71 ± 0.378	t = 3.766	<0.001
Post-OP 1 Month	0.35 ± 0.215	0.61 ± 0.417	t = 4.495	<0.001

Preoperatively, ATR astigmatism was significantly more common in Group A (temporal incision approach), observed in 48 (73.8%) patients, compared to 35 (53.8%) patients in Group B (steep-axis incision approach) (p = 0.037). Conversely, WTR astigmatism was more frequent in Group B, affecting 25 (38.5%) patients compared to 12 (18.5%) in Group A. The distribution of astigmatism types highlights a baseline variation between the groups. Postoperatively, the distribution of ATR and WTR astigmatism varied over time, but no statistically significant differences were observed between the groups at any postoperative time point (Table 5).

**Table 5:** Distribution of Astigmatism Over Time in Group A and Group B ATR, Against-the-Rule Astigmatism; WTR, With-the-Rule Astigmatism; NA, Not Applicable (No Astigmatism)

Astigmatism	Group A (n = 65)	Group B (n = 65)	Test of significance	p-value
	Frequency (%)			
Pre-OP				
ATR	48 (73.8)	35 (53.8)	χ² = 6.591	0.037
WTR	12 (18.5)	25 (38.5)		
NA	5 (7.7)	5 (7.7)		
Day 1				
ATR	42 (64.6)	40 (61.5)	χ² = 4.894	0.087
WTR	22 (33.8)	25 (38.5)		
NA	1 (1.5)	0 (0.0)		
Day 7				
ATR	33 (50.8)	43 (66.2)	χ² = 5.232	0.072
WTR	27 (41.5)	19 (29.2)		
NA	5 (7.7)	3 (4.6)		
Day 28				
ATR	30 (46.2)	47 (72.3)	χ² = 4.589	0.103
WTR	25 (38.5)	15 (23.1)		
NA	10 (15.4)	3 (4.6)		
1 Month				
ATR	30 (46.2)	47 (72.3)	χ² = 4.827	0.091
WTR	25 (38.5)	15 (23.1)		
NA	10 (15.4)	3 (4.6)		

Preoperatively, corneal astigmatism distribution was comparable between the groups (p = 0.304). By postoperative day 7, a significantly higher proportion of patients in Group A (temporal incision approach) had corneal astigmatism ≤0.5 D, seen in 42 (64.6%) patients, compared to 28 (43.1%) patients in Group B (steep-axis incision approach) (p = 0.01). At one month, Group A demonstrated significantly better astigmatic control, with 61 (93.8%) patients having astigmatism ≤0.5 D, versus 40 (61.5%) patients in Group B (p < 0.001) (Table 6).

**Table 6:** Corneal Astigmatism Distribution in Group A and Group B Over Time

Astigmatism (Corneal)	Group A (n = 65)	Group B (n = 65)	Test of significance	p-value
	Frequency (%)			
Pre-OP				
0.0 to 0.5	34 (52.3)	28 (43.1)	χ² = 2.383	0.304
0.51 to 1.0	24 (36.9)	24 (36.9)		
1.01 to 1.5	7 (10.8)	13 (20.0)		
Day 1				
0.0 to 0.5	32 (49.2)	26 (40.0)	χ² = 1.763	0.421
0.51 to 1.0	24 (36.9)	25 (38.5)		
1.01 to 1.5	9 (13.8)	14 (21.5)		
Day 7				
0.0 to 0.5	42 (64.6)	28 (43.1)	Fisher-Freeman-Halton Exact = 9.208	0.010
0.51 to 1.0	22 (33.8)	29 (44.6)		
1.01 to 1.5	1 (1.5)	8 (12.3)		
1 Month				
0.0 to 0.5	61 (93.8)	40 (61.5)	Fisher-Freeman-Halton Exact = 20.899	< 0.001
0.51 to 1.0	4 (6.2)	18 (27.7)		
1.01 to 1.5	0 (0.0)	7 (10.8)		

## Discussion

Astigmatism remains a critical determinant of visual quality following cataract surgery, with incision placement playing a pivotal role in influencing postoperative corneal curvature changes. Our study compared the SIA and refractive outcomes between the temporal incision approach (Group A) and the steep-axis incision approach (Group B) in phacoemulsification cataract surgery.

Preoperatively, UCVA and BCVA were comparable between the two groups, though BCVA was significantly better in Group B (p = 0.04). However, postoperatively, UCVA was significantly better in the temporal incision group at day 7 (p = 0.01) and at one month (p = 0.02), suggesting a more rapid visual recovery. While BCVA improved postoperatively in both groups, no significant difference was observed at any postoperative time point. These results align with studies by Soumya et al. and Hazra and Saha, which demonstrated that temporal incisions facilitate faster stabilization of refractive outcomes compared to steep-axis incisions [15,16].

The advantage of temporal incisions in early visual recovery may be attributed to their location in a more biomechanically stable region of the cornea. The superior and steep-axis incisions tend to exert greater localized stress on the corneal architecture, leading to transient fluctuations in corneal curvature and delayed stabilization of visual acuity. Previous studies by Santhanalakshmi and Srividya, and Nikose et al. support this notion, demonstrating that incisions along the steep meridian may introduce irregular astigmatic changes that persist longer postoperatively compared to temporal incisions [17,18].

One of the most significant findings in our study was the difference in SIA between the two groups. At one month, SIA was significantly lower in the temporal incision group compared to the steep-axis group (p = 0.003), indicating a more favorable refractive outcome. This aligns with prior studies, such as those by Laliwala et al. and Rathi et al., which reported that temporal incisions induce minimal corneal distortion, leading to better refractive predictability [19,20].

A key factor contributing to lower SIA in the temporal incision group is the structural integrity of the temporal cornea. The temporal limbus has relatively lower tissue resistance compared to the superior and steep meridians, allowing for more uniform wound healing and less postoperative astigmatic drift. Additionally, incisions along the steep axis are expected to neutralize pre-existing astigmatism; however, our findings suggest that this approach results in higher residual astigmatism, possibly due to greater biomechanical instability in steeper meridians. Nada et al. and Theodoulidou et al. reported similar results, highlighting that, while steep-axis incisions theoretically reduce pre-existing corneal toricity, they may also introduce greater SIA due to their anatomical location [21,22].

Corneal astigmatism was significantly lower in Group A at day 7 (p < 0.001) and at one month (p < 0.001), reinforcing the advantage of the temporal incision approach. Our results are comparable to those of Yoon et al., who found that temporal incisions led to faster corneal stability and less residual astigmatism compared to steep-axis incisions [23]. The steep meridian, being more biomechanically active, may undergo greater postoperative remodeling, contributing to higher astigmatism in the steep-axis incision group.

Preoperatively, ATR astigmatism was significantly more frequent in the temporal incision group, observed in 48 (73.8%) patients, compared to 35 (53.8%) patients in the steep-axis group (p = 0.037), while WTR astigmatism was more prevalent in the steep-axis group. This distribution is consistent with prior epidemiological studies, such as those by Tan et al. and Gupta et al., which reported a higher prevalence of ATR astigmatism in elderly populations undergoing cataract surgery [24,25].

Postoperatively, the distribution of ATR and WTR astigmatism varied over time, but no statistically significant differences were observed between the groups at any postoperative time point. This suggests that, while incision placement influences SIA magnitude, it does not significantly impact the direction of astigmatic shift. Our findings corroborate those of Patel et al., who demonstrated that, while steep-axis incisions alter SIA magnitude, they do not necessarily change the ATR/WTR shift trends in a predictable manner [26].

At one month, corneal astigmatism control was significantly better in the temporal incision group, with 61 (93.8%) patients achieving astigmatism ≤0.5 D, as compared to 40 (61.5%) patients in the steep-axis group (p < 0.001). This suggests that temporal incisions offer superior refractive predictability and stability. Similar findings were reported by Gadikar, who observed that temporal CCIs resulted in minimal postoperative astigmatic drift, leading to enhanced visual quality [27].

The stability of corneal astigmatism in the temporal incision group may be attributed to more controlled wound healing and reduced biomechanical stress on the cornea. In contrast, steep-axis incisions may induce greater local inflammatory responses and tissue remodeling, leading to unpredictable astigmatic changes over time. Studies by Vyas et al. and Tripathi et al. support this hypothesis, emphasizing that incision placement is a critical factor in long-term refractive stability [28,29].

The findings of our study reinforce the importance of incision placement in optimizing postoperative visual outcomes [30]. While steep-axis incisions are often used to address pre-existing corneal astigmatism, their higher induced astigmatism and delayed corneal stability suggest that they may not always be the best choice for all patients. Temporal incisions appear to be the preferred approach for minimizing postoperative astigmatism, ensuring faster refractive recovery, and providing greater long-term stability. For patients seeking minimal postoperative astigmatism and optimal spectacle independence, temporal incisions should be considered the first-line approach [31,32].

Limitations

While this study provides important insights into the comparative effects of temporal versus steep-axis CCIs on postoperative astigmatism, certain limitations must be acknowledged. First, the follow-up period was limited to one month, which restricts assessment of long-term refractive stability and potential astigmatic drift, as corneal remodeling may continue for several months after surgery. Second, although vector analysis of SIA was performed, the results were primarily presented as magnitudes; future work should report complete vector components, including axis changes, to enhance precision. Third, while randomization was conducted using a computer-generated table and allocation concealment was maintained by opaque, sealed envelopes, masking of the operating surgeon was not feasible. Although postoperative examiners were blinded, the absence of full masking introduces potential detection bias. Fourth, the study was limited to patients with preoperative astigmatism ≤1.5 D, which narrows external validity; the applicability of these findings to eyes with higher astigmatism or different demographic populations remains uncertain. Finally, although all surgeries were performed by a single experienced surgeon to reduce variability, this may limit generalizability to surgeons with different levels of expertise or to left-handed surgeons, given the ergonomics of temporal incisions. Potential complications, such as wound integrity issues, were rare and not extensively analyzed, representing another area for future study. Longer-term, multicenter trials with broader inclusion criteria, complete vector reporting, and standardized masking protocols are warranted to validate and extend these findings.

## Conclusions

This prospective study suggests that temporal CCIs are associated with lower early SIA and faster uncorrected visual recovery, compared with steep-axis incisions, in patients with ≤1.5 D of preoperative astigmatism. These findings support the temporal approach as a potentially advantageous technique in standard cataract surgery. However, given the short follow-up, limited masking, and restricted external validity, the results should be interpreted with caution. Larger, multicenter studies with longer-term follow-up and comprehensive vector analysis are warranted to confirm these outcomes and guide broader clinical application.

## References

[1] Sumavani V, Pakalapati P, Harinath M (2023). Awareness about senile cataracts in rural patients and their approach to tertiary eye care center for cataract surgery in Eluru district, Andhra Pradesh. Odisha J Ophthalmol.

[2] Nita M, Grzybowski A (2016). The role of the reactive oxygen species and oxidative stress in the pathomechanism of the age-related ocular diseases and other pathologies of the anterior and posterior eye segments in adults. Oxid Med Cell Longev.

[3] Upasani D, Daigavane S (2024). Phacoemulsification techniques and their effects on corneal endothelial cells and visual acuity: a review of “direct-chop” and “stop-and-chop” approaches under topical anesthesia. Cureus.

[4] Liu S, Liu J, Lin F, Yu L, Cheng C, Wang T, Zhou X (2023). Efficacy comparison between steep-Meridian incision and non-steep-Meridian incision in implantable Collamer lens surgery with low-to-moderate astigmatism. Ophthalmol Ther.

[5] Núñez MX, Henriquez MA, Escaf LJ (2019). Consensus on the management of astigmatism in cataract surgery. Clin Ophthalmol.

[6] Pattanayak S, Mathur S, Nanda AK, Subudhi BN (2022). Postoperative astigmatic considerations in manual small-incision cataract surgery - a review. Indian J Ophthalmol.

[7] Mallareddy V, Daigavane S (2024). Innovations and outcomes in astigmatism correction during cataract surgery: a comprehensive review. Cureus.

[8] Borkenstein AF, Packard R, Dhubhghaill SN, Lockington D, Donnenfeld ED, Borkenstein EM (2023). Clear corneal incision, an important step in modern cataract surgery: a review. Eye (Lond).

[9] Resch H, Pereira I, Weber S, Holzer S, Fischer G, Vass C (2015). Retinal blood vessel distribution correlates with the peripapillary retinal nerve fiber layer thickness profile as measured with GDx VCC and ECC. J Glaucoma.

[10] Hayashi K, Sasaki H, Manabe SI, Hirata A (2018). Intraocular pressure and wound state immediately after long versus short clear corneal incision cataract surgery. Jpn J Ophthalmol.

[11] Li PP, Huang YM, Cai Q, Huang LL, Song Y, Guan HJ (2019). Effects of steep-axis incision on corneal curvature in one-handed phacoemulsification. Int J Ophthalmol.

[12] Čulina K, Tomić M, Bulum T, Medić A, Šoša I, Ivanišević K, Jukić T (2024). Corneal biomechanics and other factors associated with postoperative astigmatism after cataract surgery. Life (Basel).

[13] Patel H, Congdon N, Strauss G, Lansingh C (2017). A need for standardization in visual acuity measurement. Arq Bras Oftalmol.

[14] Chylack LT Jr, Wolfe JK, Singer DM (1993). The lens opacities classification system III. Arch Ophthalmol.

[15] Soumya HV, Yadav P, Prabhakar SK (2022). Study of changes in pre-existing against-the-rule astigmatism after temporal manual small-incision cataract surgery using frown, straight, and smile incisions. Indian J Ophthalmol.

[16] Hazra S, Saha TK (2021). A comparative study of post-operative astigmatism in superior versus superotemporal scleral incisions in manual small incision cataract surgery in a tertiary care hospital. Int J Clin Exp Ophthalmol.

[17] Santhanalakshmi R, Srividya S (2019). Post-operative effect of temporal and superior approach in manual small incision cataract surgeries. MedPulse Int J Ophthalmol.

[18] Nikose AS, Saha D, Laddha PM, Patil M (2018). Surgically induced astigmatism after phacoemulsification by temporal clear corneal and superior clear corneal approach: a comparison. Clin Ophthalmol.

[19] Laliwala F, Patel S, Prajapati V, Patel L, Wanjari MB, Singhal D (2023). Comparative evaluation of astigmatic changes induced by superior and temporal corneal incisions in sutureless phacoemulsification surgery: a case series. Cureus.

[20] Rathi M, Dabas R, Verma R, Rustagi IM, Mathur S, Dhania S (2022). Comparison of surgically induced astigmatism in chevron, straight, and frown incisions in manual small-incision cataract surgery. Indian J Ophthalmol.

[21] Nada M, Rohit D, Singh SV, Khurana AK, Lochab S, Kharolia A (2022). Evaluation of scleral incisions and their effects on corneal curvature in manual small-incision cataract surgery. Indian J Ophthalmol.

[22] Theodoulidou S, Asproudis I, Athanasiadis A, Kokkinos M, Aspiotis M (2017). Comparison of surgically induced astigmatism among different surgeons performing the same incision. Int J Ophthalmol.

[23] Yoon YC, Ha M, Whang WJ (2021). Comparison of surgically induced astigmatism between anterior and total cornea in 2.2 mm steep meridian incision cataract surgery. BMC Ophthalmol.

[24] Tan QQ, Tian J, Liao X, Lin J, Wen BW, Lan CJ (2020). Impact of different clear corneal incision sizes on anterior corneal aberration for cataract surgery. Arq Bras Oftalmol.

[25] Gupta SN, Goel R, Kumar S (2022). Factors affecting surgically induced astigmatism in manual small-incision cataract surgery. Indian J Ophthalmol.

[26] Patel K, Patel V, Patel N (2020). Comparision of surgical induced astigmatism between sics superior and sics temporal. Indian J Clin Exp Ophthalmol.

[27] Gadikar AB (2020). A comparative study between superior, supero temporal and temporal incision for corneal astigmatism after small incision cataract surgery with PCIOL. MedPulse Int J Ophthalmol.

[28] Vyas VJ, Dubey S, Bhalodia HJ, Pandey A (2023). Study of comparison of surgically induced astigmatism after small incision cataract surgery and phacoemulsification surgery. Indian J Clin Exp Ophthalmol.

[29] Tripathi AK, Joshi AK, Mandlik H (2022). Comparative study of surgically induced astigmatism in superior and temporal scleral incisions in manual small-incision cataract surgery patients. Med J DY Patil Vidyapeeth.

[30] He W, Zhu X, Du Y, Yang J, Lu Y (2017). Clinical efficacy of implantation of toric intraocular lenses with different incision positions: a comparative study of steep-axis incision and non-steep-axis incision. BMC Ophthalmol.

[31] Dole K, Baheti N, Deshpande R, Kulkarni S, Shetty R, Deshpande M (2022). Comparative study of anatomical and functional recovery of eye along with patient satisfaction score after small-incision cataract surgery and phacoemulsification cataract surgery. Indian J Ophthalmol.

[32] Fathima A, Vedachalalm R, Venkatesh R, Meenakshi R, Aladin DM, Drishti C (2024). "Stamp the tunnel"- innovative tool - for perfection in manual small incision cataract surgery. Indian J Ophthalmol.

